# Multigenerational thyroid hormone resistance due to THRβ mutation

**DOI:** 10.1093/ehjcr/ytaf338

**Published:** 2025-07-17

**Authors:** Stylianos Kopanos, Joachim Feldkamp

**Affiliations:** Academic Department of Endocrinology, Diabetes and Infectiology, Klinikum Bielefeld, Medical School and University Medical Centre, East Westphalia-Lippe Bielefeld University, Teutoburgerstrasse 50, 33604, Bielefeld, Germany; Academic Department of Endocrinology, Diabetes and Infectiology, Klinikum Bielefeld, Medical School and University Medical Centre, East Westphalia-Lippe Bielefeld University, Teutoburgerstrasse 50, 33604, Bielefeld, Germany

**Keywords:** Resistance to thyroid hormone, THRβ mutation, Dilated cardiomyopathy, Refractory heart failure, Hypertrophic cardiomyopathy, Case report

## Abstract

**Background:**

Resistance to thyroid hormone (RTH) is a rare genetic disorder caused by mutations in the thyroid hormone receptors α or β (THRα, THRβ) genes, leading to impaired tissue responsiveness to thyroid hormones. While its systemic effects are well-documented, the cardiac manifestations of RTH, including hypertrophic and dilated cardiomyopathy (DCM), arrhythmias, and heart failure, are often underrecognized, particularly in cases of treatment refractory heart failure. This case report aims to highlight the importance of cardiological awareness in diagnosing and managing RTH-related cardiomyopathy.

**Case summary:**

We report the case of a 50-year-old Caucasian female with a confirmed variant c.1357C > A, p.P453T mutation in the THRβ gene, presenting with recurrent goitre, hypothyroidism, and progressive cardiovascular complications. Her clinical course was marked by episodes of angina-like symptoms, atrial fibrillation, left heart failure, and severe pulmonary oedema, eventually progressing to DCM with an ejection fraction below 30%. Despite optimal guideline-directed medical therapy, her cardiac condition deteriorated, necessitating orthotopic heart transplantation. Genetic testing confirmed the same mutation in her mother, brother, and two sons, highlighting the autosomal dominant inheritance of the disease. Thyroidectomy and lifelong levothyroxine therapy, combined with post-transplant immunosuppression, further complicated her management, underscoring the systemic interplay of RTH with cardiac function.

**Conclusion:**

This case emphasizes the rarity and clinical significance of RTH as a potential aetiology in refractory cardiac failure. Cardiologists should maintain a high index of suspicion for thyroid dysfunction in unexplained or treatment-resistant cardiomyopathy, particularly when associated with familial thyroid disorders or arrhythmias. Early diagnosis and a multidisciplinary approach involving endocrinology and cardiology are essential for improving outcomes and tailored therapeutic strategies for patients with RTH-related cardiomyopathy.

Learning points

- Mutations in the thyroid hormone receptor β (THRβ) gene can cause severe cardiomyopathy and heart failure, even after transplantation.
- Resistance to thyroid hormone should be considered in unexplained or treatment-resistant heart failure, especially with a family history of thyroid disease.
- Early multidisciplinary collaboration enables timely diagnosis and tailored therapy, improving cardiac outcomes in RTHβ patients.

## Introduction

Resistance to thyroid hormone (RTH) results from mutations that impair thyroid hormone receptor (THR) function, classified as a syndrome of reduced thyroid hormone (TH) sensitivity. It disrupts TH transport, metabolism, and receptor signalling. TH acts through receptor isoforms encoded by the THRα and THRβ genes. Loss-of-function or dominant-negative mutations in these genes impair thyroid-stimulating hormone (TSH) signalling, causing resistance.^1^

Resistance to thyroid hormone manifests with elevated free thyroxine (fT4) and free triiodothyronine (fT3) levels and unsuppressed TSH. The condition, occurring in approximately 1 in 40 000 individuals, is associated with goitre, growth delays, cognitive impairments, and cardiac arrhythmias. Diagnosis is often delayed due to its nonspecific symptoms.^2,3^

Management aims to normalize TSH and maintain an euthyroid state, with genetic counselling for affected families.^4^ Selective thyromimetic analogues can bypass receptor mutations but must be carefully monitored to avoid overactivation of TRα and cardiac issues.^5^ Here, we describe the case of a 50-year-old woman with a heterozygous THRβ c.1357C > A, p.P453T mutation, confirmed in multiple first-degree relatives, leading to severe cardiovascular complications, including hypertrophic cardiomyopathy and heart failure, ultimately requiring heart transplantation. This case emphasizes the need for incorporating thyroid-related history into cardiological evaluations.

## Summary figure

**Figure:**
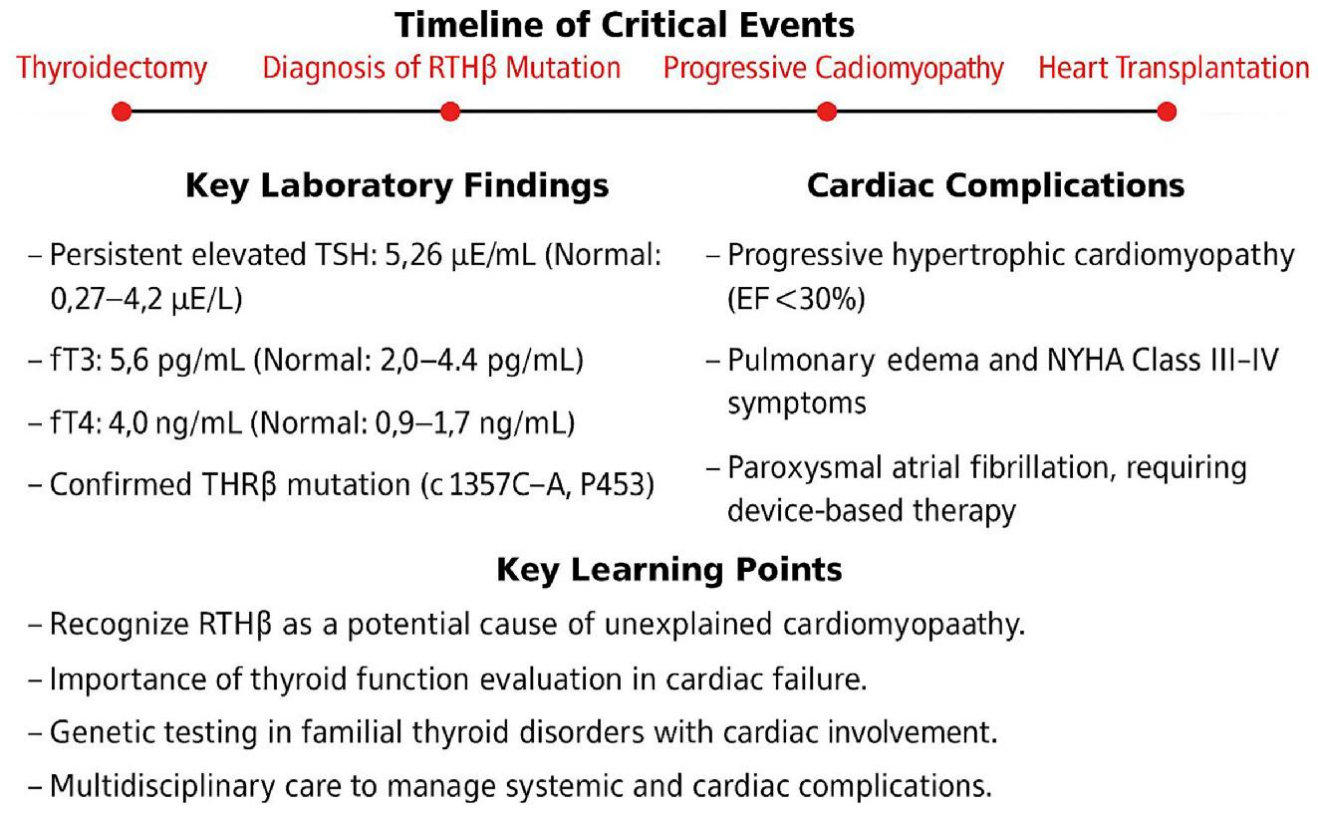


## Case presentation

A 50-year-old woman was referred for persistent hypothyroidism, presenting with fatigue, cold intolerance, weight gain, and depressive symptoms. She had undergone a thyroidectomy 2 years earlier for obstructive goitre and was on levothyroxine therapy. Physical examination was unremarkable, but family history revealed thyroid hormone resistance in her mother and brother. Her brother had restrictive cardiomyopathy, arrhythmias, and died shortly after heart transplantation, while her mother exhibited atrial fibrillation and hypertension.

Investigations revealed elevated TSH (5.26 μE/mL; normal: 0.27–4.2 μE/mL), fT3 (5.6 pg/mL; normal: 2.0–4.4 pg/mL), and fT4 (4.0 ng/mL; normal: 0.9–1.7 ng/mL) despite levothyroxine therapy. Autoimmune markers were normal, and neck ultrasound showed patchy hypoechoic parenchyma. Thyroid scintigraphy revealed 2.4% technetium uptake with no nodules. Genetic testing confirmed a heterozygous THRβ c.1357C > A, p.P453T mutation.

**Figure 1:**
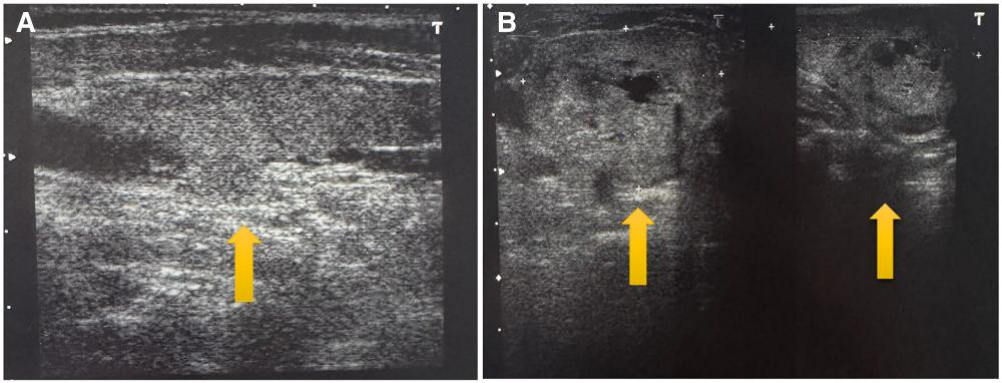
(*A*,*B*). Recurrent goitre identified liquefaction and calcifications. Images before and after thyroidectomy.

**Figure 2:**
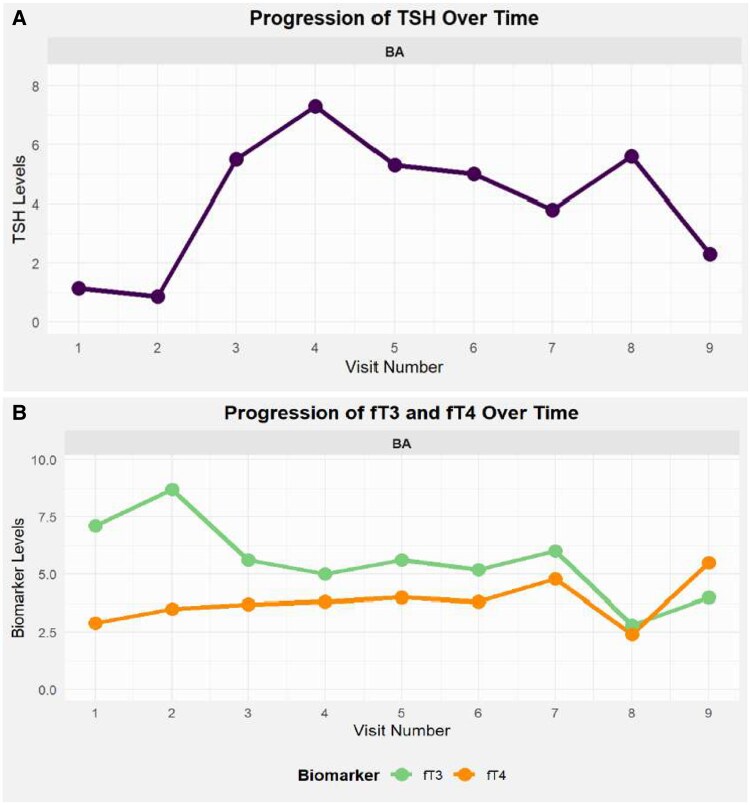
(*A*,*B*). Dynamic progression of TSH, fT3 and fT4 values over time.

Over subsequent years, the patient experienced angina-like symptoms, panic attacks, and heart failure, necessitating hospitalization for pulmonary oedema, left ventricular dysfunction (EF < 30%), and pericardial effusion. She underwent a second thyroidectomy for recurrent goitre, with an uneventful postoperative course (*Figure 1*). However, inadequate levothyroxine replacement led to weight gain, requiring initiation of liothyronine (Teatrois) at 30 mcg/day alongside levothyroxine (*Figure 2*).

Despite treatment, the patient developed progressive dilated cardiomyopathy, dialysis- dependent renal failure, and NYHA Class III-IV heart failure, leading to frequent hospitalizations. Orthotopic biatrial heart transplantation was performed, with no evidence of graft rejection. Post-transplant, thyroid hormone therapy was adjusted to levothyroxine 200 μg/day, while liothyronine was discontinued. Immunosuppressive therapy included tacrolimus, mycophenolate mofetil, and prednisolone. Secondary adrenal insufficiency was identified (ACTH: 4.7 pg/mL, cortisol: 32.2 μg/L).

The patient’s two sons were genetically tested, confirming TSHβ mutations. Both exhibited symptoms of hyperthyroidism and nodular goitre but discontinued follow-up care.

## Discussion

Thyroid hormones regulate myocardial contractility, vascular resistance, and metabolism. In RTH, particularly with THRβ mutations, hormone resistance leads to selective cardiac effects as THRα, expressed in the heart, remains functional. This causes a hyperthyroid state in cardiac tissues, promoting hypertrophy, arrhythmias, and heart failure.^6^

Excess thyroid hormone activity increases sarcoplasmic reticulum Ca²⁺ ATPase activity, predisposing patients to arrhythmias like atrial fibrillation.^7,8^ Simultaneously, impaired calcium reuptake and titin phosphorylation reduce myocardial contractility and relaxation, contributing to diastolic dysfunction and pulmonary congestion.^9,10^ Over time, these processes drive cardiac remodelling, transitioning from hypertrophic to dilated cardiomyopathy due to energy depletion and fibrosis.^11^

Genetic variability affects RTHβ presentation within families, with differences in mutation expression, severity of hormone resistance, and compensatory mechanisms.^12^ Thyroid hormone analogues like triiodothyroacetic acid bypass receptor mutations and reduce cardiac remodelling but require careful monitoring to avoid exacerbating systemic effects.^4^

Management involves balancing levothyroxine therapy while preventing cardiac overstimulation. Regular monitoring of thyroid function, echocardiography, and cardiac MRI is critical. Anti-arrhythmic therapies, beta-blockers, and RAAS inhibitors are often needed to stabilize cardiac status. In severe cases like this, heart transplantation offers the only viable solution but introduces challenges, including immunosuppressive therapy interactions with thyroid hormones.^13,14^

## Conclusion

This case highlights the significant cardiovascular impact of RTHβ, emphasizing the need for its consideration in treatment-resistant cardiomyopathy. Importantly, it underlines the critical role of early diagnosis: prompt identification of thyroid hormone resistance could enable earlier therapeutic interventions, potentially preventing the development of severe cardiac remodelling, arrhythmias, and heart failure.

Incorporating routine thyroid function screening and detailed family history analysis into the diagnostic workup of unexplained cardiomyopathy could significantly improve outcomes. Recognizing elevated thyroid hormone levels with non-suppressed TSH should prompt genetic evaluation for RTHβ, particularly in the presence of cardiac symptoms. Early multidisciplinary intervention combining endocrine, cardiological, and genetic expertise can prevent disease progression and reduce the need for advanced interventions such as heart transplantation.

Increased clinical awareness of this underdiagnosed syndrome will foster earlier detection, personalized management, and better long-term prognoses for affected patients.

## Data Availability

Data supporting these case reports are stored locally in our hospital and can be provided on request.

## Lead author biography

Dr. Stylianos Kopanos is a physician specializing in Internal Medicine, Endocrinology, and Diabetology, currently pursuing his training at Klinikum Bielefeld, UK OWL, Germany. He is current a PhD student at Bielefeld University and holds three Master of Science (MSc) degrees in Medical Research Methodology, Diabetes & Obesity, and Drug Development from leading Greek universities. Dr. Kopanos has extensive clinical and research experience, with a focus on endocrinological disorders and metabolic diseases. He has contributed to multiple international publications and presentations and actively participates in clinical trials and meta-analyses.
